# Infant formula fatty acid profile following microwave heating

**DOI:** 10.1371/journal.pone.0237391

**Published:** 2020-08-28

**Authors:** Jan Krzysztof Nowak, Szymon Kurek, Jarosław Walkowiak, Sławomira Drzymała-Czyż

**Affiliations:** 1 Department of Pediatric Gastroenterology and Metabolic Diseases, Poznan University of Medical Sciences, Poznan, Poland; 2 Department of Bromatology, Poznan University of Medical Sciences, Poznań, Poland; Higher Institute of Applied Sciences and Technology of Gabes University of Gabes, TUNISIA

## Abstract

Microwave heating of foods may alter their content. Yet, parents commonly heat infant formula in the microwave oven. The study aimed at understanding whether microwave heating of formula changes its fatty acid (FA) composition. Portions of infant formula were prepared and divided in three parts: control (sampled twice: at the start and after 30 minutes), microwave (sampled twice: after reaching 37°C and 50°C), and water bath (sampled twice: after reaching 37°C and 50°C). In thus obtained samples, a total profile of 25 FA was assessed using gas chromatography-mass spectrometry. Overall, fourteen portions were prepared, which were sampled 84 times yielding 2075 individual total FA level measurements. Few differences were identified between the microwave, control, and water bath groups. Microwave warming to 37°C was associated with increases of C12 (median increased by +0.40%, p = 0.0063), C14 (+0.05%, p = 0.0091), and C4 content (+6.90%, p = 0.0131). Microwaving up to 50°C slightly decreased C16:1trans (-5.00%, p = 0.0463) and C18:2trans1 (-5.13%, p = 0.0231). A paired comparison of pooled control and microwaved samples revealed increases in C12 (+0.18%, p = 0.0490) as well as a loss of C18:2trans1 (also -5.13%, p = 0.0073) and C18:2trans3 (-5.56%, p = 0.0042) after microwave treatment. C18:2trans1 (-2.63%, p = 0.0132) and C18:3trans1 (-2.26%, p = 0.0434) were lower in microwaved samples compared with the water bath. A slightly lower C18:2 content was found in the water bath samples than in the control groups (-0.11%, p = 0.0430). None of these differences would remain significant after a correction for multiple comparisons. Microwave heating of infant formula to 37°C or 50°C might marginally alter its total FA profile. Further studies are required to determine whether it alters the rate of free radical formation.

## Introduction

Despite the evident benefits of breastfeeding, many infants in developed countries are formula-fed [1]. In the United Kingdom this concerns 79% of six-week-old infants [2], in Poland 59%-68% of four-month-old children [3, 4]. Australian Health Ministers’ Advisory Council reported in 2018 that two thirds of infants received formula by four months of age. The rates of exclusive breastfeeding decrease with age, e.g., in Poland it is continued in less than 17% after six months [5]. Parents often save the milk for later and reheat when required [6]. One of the more popular methods to do so is microwaving [7]. Between 20% and 66% of caregivers use the microwave oven to bring the formula to the desired temperature [8–10].

Microwaving was shown to alter the fatty acids profile in selected foods. In the case of eggs cooked to a high temperature it may lower oleic acid content by almost a half and increase palmitic acid level by a fourth [11]. Little is known about the influence of microwave heating on fatty acid (FA) composition of dairy products since the studies are scarce and the number of assessed FA was small. It seems that microwaving might increase trans FA in overheated (90 seconds) milk by a third and reduce the conjugated linoleic acid in cheese by a fifth [12, 13]. The effects of microwaving breast milk were assessed in one study, which did not find any changes in the only two investigated FA: linoleic and alpha-linoleic [14].

FA influence infant development through regulation of cellular functions, intra- and extracellular signalling, and epigenetic regulation [15]. FA composition of infant formula is a relevant topic of study because it may influence child development. The presence of oleic acid (C18:1) and linoleic acid (C18:2) facilitates triglyceride digestion and absorption, thus maximizing energy supply. Saturated fatty acids, which constitute over a fifth of the formula, also provide easily accessible nutritional value and are available from the mother’s diet. There are, however, FAs with special functions. Docosahexaenoic acid (C22:6), which cannot be synthesized by infants, plays an important role in the development of the brain and the retina [16]. Trans FAs, especially trans isomers of the oleic acid (C18:1), which typically originate from highly-processed foods, might negatively impact growth by inhibiting desaturation and elongation of the linoleic acid (C18:2) to arachidonic acid (C20:4) as well as the transition of alpha-linoleic acid (C18:3) to docosahexaenoic acid (C22:6) [17]. In fact, vaccenic acid (C18:1n-7) is the only natural trans FA in the breastmilk as a result of consumption of ruminant-derived products [18]. It might reduce the concentration of proatherogenic lipid fractions and slow lipogenesis, and was suggested to confer a number of other benefits [19]. Overall, the adequate FA profile of infant formula is increasingly recognized as a crucial early determinant of long-term metabolic health [20].

Fleddermann et al. demonstrated that changes of the composition of the formula, including the FA profile, are reflected in the child’s metabolic profile and development [21]. However, the effects of microwaving on FA composition of infant formula have not been investigated. Moreover, there are few investigations simulating real-life conditions of milk heating. Therefore we hypothesized that microwave heating of infant formula to 37°C or 50°C leads to changes in the total FA profile.

## Materials and methods

### Study design

**Fig 1:**
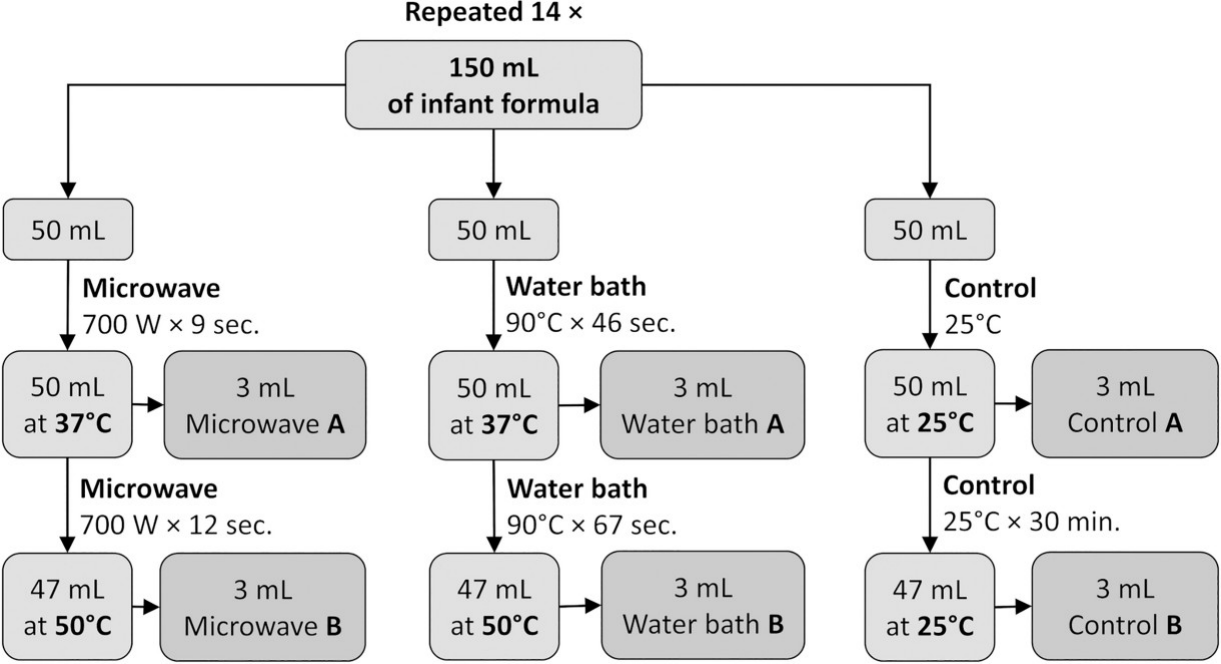
Study design.

A first infant formula based on cow’s milk (NAN Optipro Plus 1; Nestlé, Vevey, Switzerland) was prepared according to manufacturer’s instructions (150 mL water + 5 scoops). From each portion six samples of 3 mL were obtained as described below, each time preceded by shaking and temperature measurement (Fig 1).

After initial sampling (control A), directly following the preparation, the formula was left for 30 minutes at room temperature (24–25°C); then another sample was taken (control B). Meanwhile, 50 mL of formula was microwaved for 9 seconds at full power in order to reach the temperature of 37°C (microwave A) and then for further 12 seconds to reach 50°C (microwave B) (700 W at 2450 MHz, turntable plate; Alaska MW 2005, SIG GmbH, Düsseldorf, Germany). A portion of the formula was placed in a water bath (90°C) for 46 seconds, which heated the milk to 37°C (water bath A), and then for further 67 seconds for the temperature to reach 50°C (water bath B). The temperature was measured using the same glass laboratory thermometer.

### Fatty acid profile assessment

Total FAs were assessed using a modified Gotlieb's method [22]. The samples were prepared for processing by the addition of 0.3 mL of concentrated H_2_SO_4_ and maintaining 65°C for 15 minutes. Then, 3 mL of ethanol (99%) were added and the sample was vortexed. The extraction was performed twice with 2 mL of n-hexane and centrifugation at 2300 rpm for 10 minutes. The supernatant was dried in nitrogen at room temperature. All the samples were frozen without delay at -20°C. Ten μL of the extract were vortexed with 20 μL of 2N methanolic KOH. Then, 1 mL of n-hexane was added, the sample was vortexed and left for 5 minutes at room temperature. For the analysis, 200 μL of the extract were placed in the chromatographic vial. Gas chromatography with mass spectrometry was conducted using Agilent 7890 series II and 5975C (Agilent Technologies, Santa Clara, CA, USA) with BPX70 column (SGE Analytical Science, Ringwood, Australia). Peaks were analyzed using MSD ChemStation (Agilent Technologies).

### Data analysis

Statistical analyses were conducted using Python 2.7.12 (Python Software Foundation, Delaware, USA) with NumPy 1.11.0, Pandas 0.17.1, and SciPy 0.17. The Shapiro-Wilk test was used to test for normality and the Wilcoxon test was employed for paired data analyses. The results are presented as medians [1^st^-3^rd^ quartiles] unless specified otherwise. A relative difference between medians expressed as a percentage is provided in the results section. While the significance threshold α was set at 0.05, β was assumed to be 0.10 in order to evade type II error (failing to identify FA alterations), which should be minimized in this study. Since 28 comparisons were made when two profiles are compared (25 FA along with three spectra of uncertain origin labelled X1, X2, and X3), the Holm-Bonferroni corrected alpha level would be 0.0018. However, to reduce type II error, we present all the results with the standard p value < 0.05. The reasons for this are provided in the discussion. GPower 3.1.9.2 (University of Düsseldorf, Germany) was used to perform the post-hoc sample size calculation. Considering coefficients of variation in the studied groups and the sample size of 14 the median difference detectable with the power of > 90% was of 3.4% [1.4–11.5%].

### Ethics

This study did not involve human subjects and therefore did not require bioethical committee approval.

## Results

**Fig 2:**
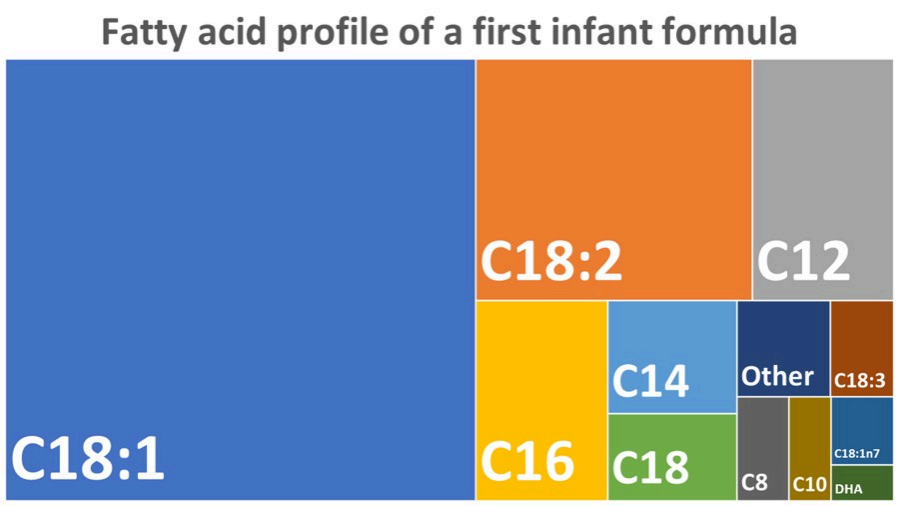
The total FA composition of the investigated first infant formula as determined using gas chromatography-mass spectrometry, based on data from the analysis of unheated samples (control A).

Overall, despite including 2075 data points (full dataset: S1 Table), few differences were identified between the microwave, control, and water bath groups, of which all were minimal and became statistically insignificant after the correction for multiple testing. The proportions of total FA in the investigated first infant formula are presented in Fig 2.

Microwaving to 37°C was associated with increases of lauric acid (C12, +0.40%), myristic acid (C14, +0.05%), and butyric acid content (C4, +6.90%) (Table 1). Analogous changes were not observed with microwaving up to 50°C, which in turn decreased C16:1trans (-5.00%) and C18:2trans1 (-5.13%) (Table 2; S2 Table).

**Table 1:** Mean (±SD) relative abundance of fatty acids in microwaved infant formula (37°C) compared with control samples.

Fatty acid	Control A (%)	Microwave A (%)	p
C18:1	53.029 ± 0.237	52.890 ± 0.224	NS
C18:2	17.048 ± 0.101	16.993 ± 0.080	NS
C12	8.641 ± 0.141	8.746 ± 0.131	0.0063
C16	6.716 ± 0.054	6.741 ± 0.024	NS
C14	3.731 ± 0.063	3.767 ± 0.041	0.0091
C18	2.853 ± 0.029	2.860 ± 0.015	NS
C18:3	1.557 ± 0.012	1.551 ± 0.006	NS
C8	1.359 ± 0.020	1.375 ± 0.027	NS
C10	1.111 ± 0.019	1.130 ± 0.033	NS
C18:1n7	1.091 ± 0.014	1.087 ± 0.005	NS
C22:6	0.563 ± 0.033	0.574 ± 0.028	NS
20:5	0.156 ± 0.003	0.156 ± 0.003	NS
C4:0	0.029 ± 0.001	0.031 ± 0.002	0.0131
C16:1trans	0.040 ± 0.003	0.040 ± 0.007	NS
C18:2trans1	0.039 ± 0.003	0.037 ± 0.002	NS

Fatty acids representing > 0.5% of total are presented, as well as the eicosapentaenoic acid (EPA) and other fatty acids, for which differences were found. The complete data are presented in S2 Table.

**Table 2:** Mean (±SD) relative abundance of fatty acids in microwaved infant formula (50°C) compared with control samples.

Fatty acid	Control B (%)	Microwave B (%)	p
C18:1	52.958 ± 0.196	52.906 ± 0.301	NS
C18:2	17.006 ± 0.065	17.020 ± 0.093	NS
C12	8.668 ± 0.093	8.708 ± 0.146	NS
C16	6.735 ± 0.030	6.739 ± 0.040	NS
C14	3.745 ± 0.041	3.758 ± 0.051	NS
C18	2.863 ± 0.020	2.863 ± 0.019	NS
C18:3	1.552 ± 0.007	1.559 ± 0.014	NS
C8	1.366 ± 0.019	1.371 ± 0.024	NS
C10	1.114 ± 0.014	1.119 ± 0.020	NS
C18:1n7	1.091 ± 0.005	1.090 ± 0.015	NS
C22:6	0.573 ± 0.031	0.565 ± 0.012	NS
C20:5	0.155 ± 0.003	0.156 ± 0.003	NS
C4:0	0.030 ± 0.001	0.029 ± 0.001	NS
C16:1trans	0.044 ± 0.012	0.038 ± 0.002	0.0463
C18:2trans1	0.040 ± 0.004	0.037 ± 0.002	0.0231

Fatty acids representing > 0.5% of total are presented, as well as the eicosapentaenoic acid (EPA) and other fatty acids, for which differences were found. The complete data are presented in S2 Table.

A paired comparison of pooled control (A+B) and microwaved (A+B) samples revealed increases in lauric acid (C12, +0.18%) as well as loss of C18:2trans1 (-5.13%) and C18:2trans3 (-5.56%) after microwave treatment (S3 Table). C18:2trans1 (-2.63%) and also C18:3trans1 (-2.27%) were lower in microwaved samples compared with the water bath. A slightly lower C18:2 content (-0.11%) was found in the pooled water bath samples (A+B) than in the control (A+B) groups. Comparison of water bath A (37°C) with control A as well as of water bath B (50°C) with control B is presented in S2 Table.

The median temperatures in all the groups were achieved according to the plan: 25°C (control A and B), 37°C (microwave A, water bath A), 50°C (microwave B and water bath B) (S4 Table).

## Discussion

This is the first study to investigate changes of total FA profile introduced by infant formula heating. We have found evidence of at most minimal alterations brought about by the use of microwave and water bath. Below we motivate the selection of formula and sample volume, further comment on the results and discuss limitations of the above experiment.

Thermic oxidation of lipids reduces the quality of food by reducing the content of beneficial constituents and introducing potentially toxic compounds. Polyunsaturated FA are especially vulnerable to this process [23]. Sunflower, palm and soy oils as well as olive oil are more susceptible to oxidation during microwave treatment exceeding 30 minutes at full power than with some traditional heating methods [24]. One of our hypotheses was that the longest FA would be more sensitive to microwaving.

For this reason NAN Optipro Plus 1 was chosen: among all the formulas available on the Polish market it boasts the largest docosahexaenoic acid concentration at 17.4 mg/100 mL as declared by the manufacturer and consistent with proportions found in this study. An additional factor supporting the choice of a first formula was the inexperience of new parents, who might commit more errors during its preparation. As Labiner-Wolfe et al. observed, 77% of mothers do not receive any advice on formula mixing from healthcare workers, 35% of them use the microwave, and 6% do not dispose of the left-over milk which was standing for longer than 2 hours [25].

The portion size of 50 mL is small, but reflects the situation of new parents. They care for a neonate who usually requires between 100 and 120 mL of formula per feeding which might be prolonged or interrupted by sleep. The remaining milk may be reheated. Of note, samples as small as 1 mL were used in a study that demonstrated the loss of anti-infective milk properties after microwaving at high temperatures [26]. On the other hand, microwave heating of milk might be used to prevent CMV infection, on par with Holder pasteurization [27].

This work suggests that in the above-described neonatal scenario, microwaving might lead to at most minimal and selective increase of saturated FA (butyric C4:0, lauric C12:0, and myristic acid C14:0) and concomitant reduction of trans FA (palmitoleic acid C16:1trans; C18:2trans1, C18:2trans3). These data could not be compared with the literature because of the lack of analogous work on infant formula. Moreover, the scale of the observed changes was negligible, and they were not significant after a comparison for multiple testing. It is not surprising that water bath might slightly alter FA as well since heating, regardless of the method employed, may alter food composition.

Nevertheless, it should be stressed that oxidized FA were not measured directly and may have very well increased. The total FA assessment method by transesterification could not detect changes in FA content in lipid fractions. Microwaving milk might also impact non-lipid components including protein or carbohydrates (e.g., Maillard reactions). More research on this topic could yield arguments promoting parental adherence to the recommended formula preparation procedure. A systematic review of the techniques of human milk heating was recently presented by Wesolowska et al. [28].

The electromagnetic radiation generated by consumer microwaves has the wavelength of 2450 MHz, which is specifically adapted for dielectric heating of liquid water. Nevertheless, the microwave oven design could influence FA oxidation. Prevention of standing wave patterns is crucial in this aspect, since it would precipitate uneven cooking and favorize the appearance of extreme local maxima of temperature and thus FA oxidation. Therefore, microwaves include the rotating dish or a stirrer fan. A more even distribution of the electromagnetic waves also helps prevent mouth burns, which nevertheless still present a real risk for the infant. Any heating of milk in a plastic bottle containing bisphenols, phthalates, or other similar additives, could also potentially lead to their release and thus have detrimental impact on child’s health. We have investigated microwave heating at full power. However, alternative settings, which usually involve intermittent magnetron activation, might affect FAs in a different way.

Correction for multiple testing is uncommon in FA research [29]. The results are presented with the standard threshold of 0.05 as well as with the Holm-Bonferroni correction, which made all the results insignificant. Multiple testing corrections are well-known to increase the ratio of false negative results. However, this topic concerns safety and therefore minimizing false negatives is crucial. Therefore, both raw p values and the Holm-Bonferroni threshold are presented so as to give the reader the opportunity to interpret the data independently.

One of the limitations of this study is the quantity of milk heated. Larger volume might allow for more pronounced changes to be found. The scope of this work was narrowed to the assessment of FA, without considering other nutrients or free radical formation. The FA assessment method required moderate heating of the samples, which could introduce a systematic bias to the measurements, but should not influence the main results. Efforts were made to minimize the potential influence of a number of factors including formula concentration and the time of processing individual samples within a batch. Limited variation within the data indicates that this approach was successful. The main strengths of the study are the precision and comprehensiveness of FA assessment as well as a faithful representation of real-life conditions. Another advantage is the study of a commercially available FA composition of a first infant formula.

In conclusion, microwave heating of infant formula to 37°C or 50°C might marginally alter its total FA profile. Further studies are required to determine whether it alters the rate of free radical formation.

## Supporting information

S1 Table Full dataset. Relative abundance of various fatty acids in samples, which were left untreated, microwaved to 37 degrees Celsius (A) and 50 degrees Celsius (B), as well as heated in water bath to 37 degrees Celsius (A) and 50 degrees Celsius (B).
(XLSX)

S2 Table Results of paired comparisons of the relative abundance of fatty acids in samples, which were left untreated, microwaved, and heated in a water bath. The achieved temperatures were 37 degrees Celsius (A) and 50 degrees Celsius (B). The Wilcoxon test was used.
(XLSX)

S3 Table Matched comparisons of the relative abundance of fatty acids in all the samples, which were left untreated, microwaved or heated in a water bath. (XLSX)

S4 Table Sample temperatures. (XLSX)
